# PDZ Domain-Mediated Interactions of G Protein-Coupled Receptors with Postsynaptic Density Protein 95: Quantitative Characterization of Interactions

**DOI:** 10.1371/journal.pone.0063352

**Published:** 2013-05-14

**Authors:** Thor C. Møller, Volker F. Wirth, Nina I. Roberts, Julia Bender, Anders Bach, Birgitte P. S. Jacky, Kristian Strømgaard, Jan M. Deussing, Thue W. Schwartz, Karen L. Martinez

**Affiliations:** 1 Department of Chemistry & Nano-Science Center, University of Copenhagen, Copenhagen, Denmark; 2 Department of Neuroscience and Pharmacology & Novo Nordisk Foundation Center for Basic Metabolic Research, University of Copenhagen, Copenhagen, Denmark; 3 Department of Drug Design and Pharmacology, University of Copenhagen, Copenhagen, Denmark; 4 Molecular Neurogenetics, Max Planck Institute of Psychiatry, Munich, Germany; 5 Clinical Cooperation Group Molecular Neurogenetics, Institute of Developmental Genetics, Helmholtz Center Munich, Munich, Germany; Harvard Medical School, United States of America

## Abstract

G protein-coupled receptors (GPCRs) constitute the largest family of membrane proteins in the human genome. Their signaling is regulated by scaffold proteins containing PDZ domains, but although these interactions are important for GPCR function, they are still poorly understood. We here present a quantitative characterization of the kinetics and affinity of interactions between GPCRs and one of the best characterized PDZ scaffold proteins, postsynaptic density protein 95 (PSD-95), using fluorescence polarization (FP) and surface plasmon resonance (SPR). By comparing these *in vitro* findings with colocalization of the full-length proteins in cells and with previous studies, we suggest that the range of relevant interactions might extend to interactions with *K*_i_ = 450 µM in the *in vitro* assays. Within this range, we identify novel PSD-95 interactions with the chemokine receptor CXCR2, the neuropeptide Y receptor Y_2_, and four of the somatostatin receptors (SSTRs). The interaction with SSTR1 was further investigated in mouse hippocampal neurons, where we found a clear colocalization between the endogenously expressed proteins, indicating a potential for further investigation of the role of this interaction. The approach can easily be transferred to other receptors and scaffold proteins and this could help accelerate the discovery and quantitative characterization of GPCR–PDZ interactions.

## Introduction

G protein-coupled receptors (GPCRs), also called seven-transmembrane receptors, constitute the largest family of membrane proteins in the human genome [1]. Their signaling is mediated by numerous proteins and is still not completely elucidated. This network of proteins is organized and regulated by scaffold proteins forming several transient interactions with GPCRs and cytosolic signaling proteins [2]–[5]. In this way, scaffold proteins influence several aspects of GPCR signaling, such as desensitization, internalization, and post-endocytic sorting; the understanding of these interactions is therefore important to understand cell signaling.

PDZ domains are among the most common protein interaction domains in scaffold proteins: More than 150 human proteins contain one or more of these 80–100 amino acid (aa) domains, often in combination with other protein interaction domains [6]. PDZ domains typically form weak transient complexes (i.e. complexes that readily dissociate) with C-terminal short linear motifs [7].

The scaffold protein postsynaptic density protein 95 (PSD-95) is one of the major components of the postsynaptic density of excitatory glutamatergic synapses, where it organizes signaling complexes close to the membrane [6]. PSD-95 contains a Src homology 3 (SH3)–guanylate kinase-like (GK) supramodule and three PDZ domains that bind to class I PDZ motifs (–X–S/T–X–Φ–COO^−^, where X is any aa, and Φ is a bulky hydrophobic aa [F, I, L, M, V, W]). The first two PDZ domains in PSD-95 are separated by only 5 aa and constitute a supramodule that generates larger clusters of Kv1.4 channels than a mutant with a 14 aa linker between the two domains [8].

A few GPCRs have been shown to interact specifically with the PDZ domains of PSD-95 with various effects on GPCR signaling; for example, PSD-95 was shown to be important for the dendritic localization of the 5-hydroxytryptamine receptor 2A (5-HTR_2A_) in cortical pyramidal neurons [9] and to increase the agonist efficacy and decrease the agonist mediated internalization of 5-HTR_2A_ in HEK293 cells [10]. In the case of the β_1_-adrenergic receptor (β_1_AR), PSD-95 was shown to decrease agonist stimulated internalization of the receptor and to facilitate interaction between β_1_AR and the NMDA receptor [11].

Although many GPCR–PDZ interactions are now known, most have only been described by qualitative methods, such as yeast two-hybrid [11], protein arrays [12], pull-down [2], co-immunoprecipitation [10], [13], and affinity purification [13], [14]. To predict how a cell behaves under different conditions, it is necessary to describe the interactions quantitatively, i.e. in terms of affinity and kinetics, and to know how a protein interacts with the individual domains for multidomain proteins, such as scaffold proteins. Whereas the domain specificity is known qualitatively for some GPCR–PSD-95 interactions [11], [14]–[16], the affinity and kinetics is not known for any of them.

Here, we determine the affinity, kinetics and domain preference for the interactions between PSD-95 and a wide range of GPCRs. As it is inherently difficult to perform quantitative measurements and to obtain information about the molecular details of an interaction in the complex environment of living cells, we have taken a reductionist approach; we used synthetic peptides mimicking the C-tails of GPCRs and purified, isolated PDZ domains from PSD-95 and characterized them *in vitro* by fluorescence polarization (FP) and surface plasmon resonance (SPR). We further show that the affinities measured *in vitro* are consistent with colocalization of full-length GPCRs and PSD-95 in HEK293 cells and in hippocampal neurons.

## Results

### FP Characterization of GPCR–PDZ Interactions

We measured the *in vitro* affinity of interactions between a wide range of GPCR C-terminal tails (C-tails) and the three isolated PDZ domains composing PSD-95. A library of 34 synthetic peptides mimicking the C-terminal 10 aa of GPCR C-tails (25 of them with a class I PDZ motif) was constructed for this purpose (Table S1). The affinities were measured by FP, which is a solution-based technique that does not require separation of bound from unbound ligand. This method has previously proved suitable for measuring the affinity of PDZ domain interactions [17]–[19].

**Figure 1:**
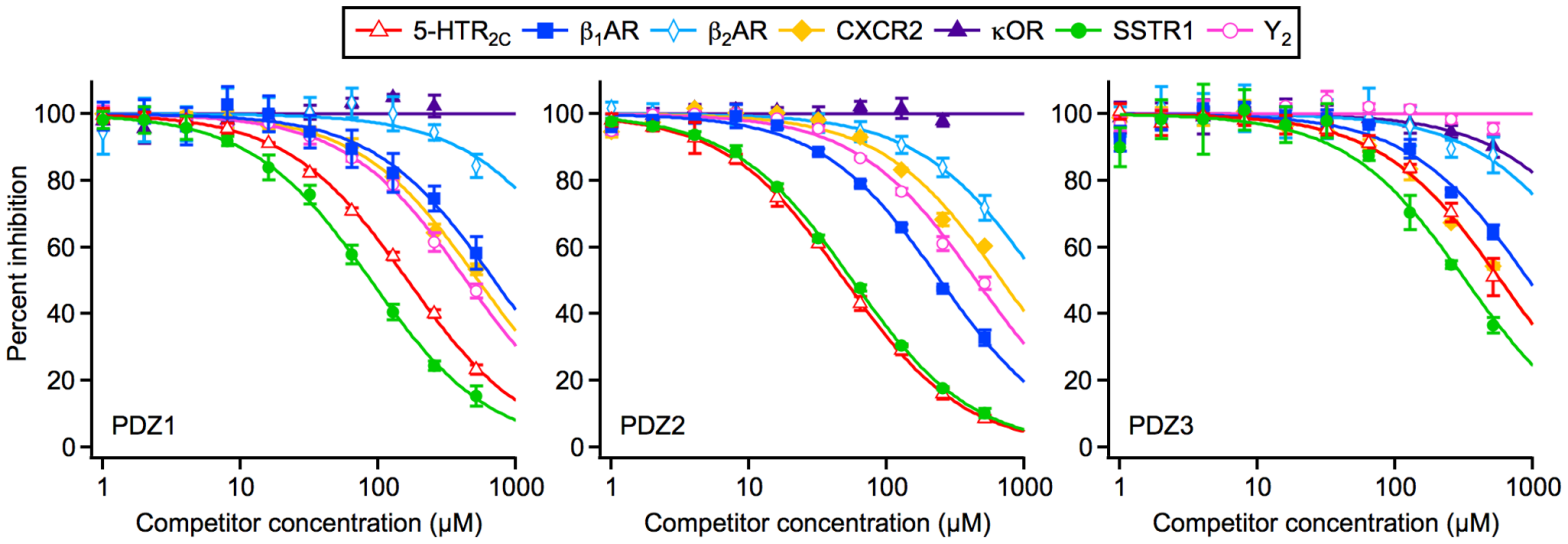
Representative FP competition curves. Binding of 1–512 µM GPCR C-tail peptides to a fixed concentration of PSD-95 PDZ1 (left panel), PDZ2 (middle panel) or PDZ3 (right panel) and Cy5-labeled probe. The data points are averages of three independent measurements, and the error bars represent the standard error of the mean. The solid lines are the fitted curves.

We used a competition set-up, where a constant concentration of isolated PSD-95 PDZ domain and a labeled reference ligand was titrated with unlabeled peptides from the GPCR C-tail library, typically within a 1–512 µM concentration range, and the resulting curves were fitted to obtain the IC_50_, which was used to calculate the *K*_i_ (Figure 1). Three Cy5-labeled peptides with well-documented interactions with PSD-95 were used as reference ligands for the competition assay: Cy5-KIF1Bα was used for PDZ1 [20], Cy5-GluN2B (formerly known as NR2B) was used for PDZ2 and PDZ1-2 [21], and Cy5-CRIPT was used for PDZ3 [22] (Table S1). Competition with unlabeled versions of the reference ligands was used to validate the FP assay by comparison with the literature (Table S2).

We determined the *K*_i_ for 7 uncharacterized known binders and 8 potential new binders (Table 1) and identified 19 non-binders (Table S3). Most of the receptors that had previously been shown to interact with PSD-95 in cells or tissues were found among the strongest interactions (considering only the interaction with the strongest binding PDZ domain for each GPCR C-tail); specifically, 5-HTR_2A_, 5-HTR_2C_, β_1_AR, and BAI1 receptor interacted with PDZ2 with *K*_i_ around 100 µM or lower. One of the previously untested peptides, human SSTR1 (hSSTR1), interacted with PDZ2 with a *K*_i_ below 100 µM, making it a good candidate for studies in cells.

**Table 1:** *K*_i_ values for GPCR C-tail interactions with the PSD-95 PDZ domains determined by FP.

			*K*_i_ (µM)a,b			
Competitor	Species	Family	PDZ1	PDZ2	PDZ3	Previously known
5-HTR_2A_	Human	5-Hydroxytryptamine receptors	160±4	46±0.2^c^	NA	[9], [10]
5-HTR_2C_	Human	5-Hydroxytryptamine receptors	100±0.1^c^	26±0.4^c^	370±40	[13], [15]
β_1_AR	Human	Adrenergic receptors	430±47	120±20^c^	360±82^c^	[11]
β_2_AR	Human	Adrenergic receptors	NA	720±15^c^	NA	[16]
CXCR2	Human	Chemokine receptors	280±25^c^	370±53	370±39	
BAI1	Human	Class B Orphans	63±6.3^c^	29±1.9^c^	790±93	[18]
mGlu_1(a)_	Human	Metabotropic glutamate receptors	830±82	NA	NA	
mGlu_7(a)_	Human	Metabotropic glutamate receptors	610±51	NA	NA	
Y_2_	Human	Neuropeptide Y receptors	270±22	230±14^c^	NA	
SSTR1	Human	Somatostatin receptors	50±3.2^c^	28±2^c^	200±34	
SSTR1	Mouse	Somatostatin receptors	270±35	90±14^c^	NA	[14] d
SSTR2A	Human	Somatostatin receptors	620±106	230±2^c^	NA	
SSTR3	Human	Somatostatin receptors	610±125	NA	280±5^c^	
SSTR4	Human	Somatostatin receptors	750±80	450±58	600±67	
SSTR4	Mouse	Somatostatin receptors	NA	420±52	NA	[14]

a The shown data are *K*_i_ ± fitting error, unless otherwise noted.

b NA, no affinity, defined as a *K*_i_ value above 1000 µM.

c Shown data are mean *K*_i_ ± standard error of the mean from two or more independent experiments.

d Only shown to interact with PSD-95 *in vitro*.

Most of the newly identified interactions had *K*_i_ in the 200–450 µM range, specifically the chemokine receptor CXCR2, the neuropeptide Y receptor Y_2_, SSTR2A, SSTR3, and hSSTR4, whereas only one of the interactions identified in the literature, mSSTR4, was found in this range. It is thus not clear from the current literature whether these receptors are likely to interact with PSD-95 in cells.

### Kinetics of GPCR–PDZ Interactions

To confirm the affinities measured by FP using an orthogonal method and to get information about the kinetics of the GPCR–PDZ interactions, we used SPR. This label-free, surface sensitive technique measures the binding of a ligand to an immobilized protein as a function of time based on refractive index changes. In order to characterize the interactions between PSD-95 and GPCR C-tails, we immobilized the three PSD-95 PDZ domains on separate carboxymethyl dextran surfaces and confirmed the function of the immobilized PDZ domains with the same reference ligands as in the FP assay (Table S4): KIF1Bα for PDZ1 [20], KIF1Bα and GluN2B for PDZ2 [20], [21], and CRIPT for PDZ3 [22].

**Figure 2:**
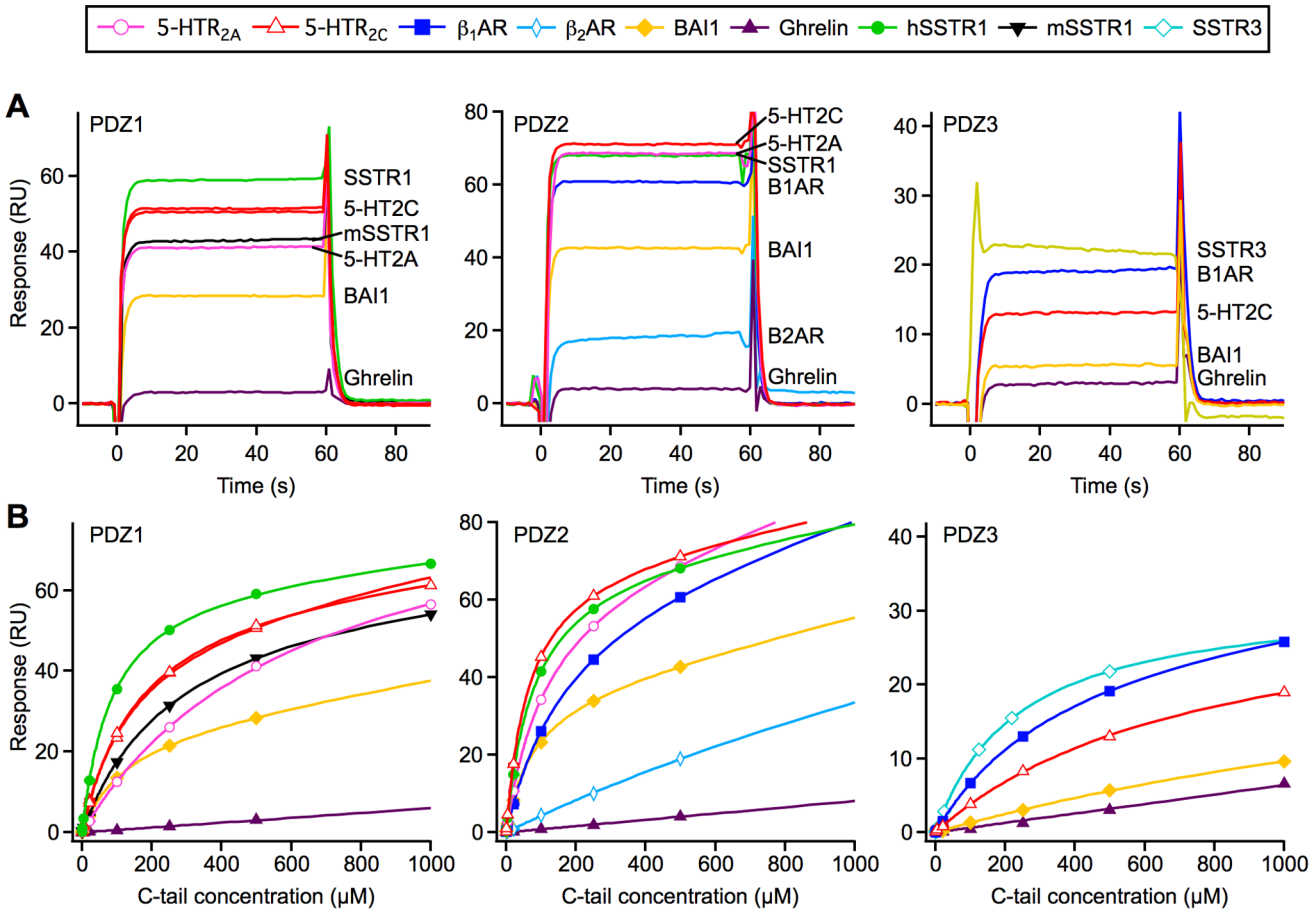
Time-resolved binding of GPCR C-tails to the PSD-95 PDZ domains. Binding of GPCR C-tail peptides to immobilized PSD-95 PDZ1 (left panel), PDZ2 (middle panel), or PDZ3 (right panel) monitored by SPR. (A) Time-resolved binding of 500 µM GPCR C-tail peptides. (B) Steady-state responses and the corresponding fitted curves (solid lines). The curves are reference and blank subtracted.

For each of the PSD-95 PDZ domains, we determined the *K*_d_ and the stability of the complexes for selected GPCR C-tail peptides. The residence time τ ( = 1/*k*_d_) was used as a convenient measure of the stability of the complexes [23]. For all three PDZ domains, we observed a clear interaction with the well-characterized binder 5-HTR_2C_, whereas a receptor without a PDZ motif, the ghrelin receptor (RAWTESSINT–COO^–^), did not interact (Figure 2A). The *K*_d_ determined from the steady-state responses of a range of C-tail peptide concentrations (Figure 2B and Table S4) correlated well with the *K*_i_ values obtained by FP (Figure S1).

For all combinations of PDZ domains and GPCR C-tail peptides, we observed dissociation of the complexes within the first second of the dissociation phase (Figure 2A) (faster than the time-resolution of the instrument), meaning that τ is less than 1 s (*k*_d_ >1 s^−1^). This implies that the GPCR–PDZ complexes are very transient, which is in agreement with previous work [24].

### Domain Preference and Peptide Selectivity

**Figure 3:**
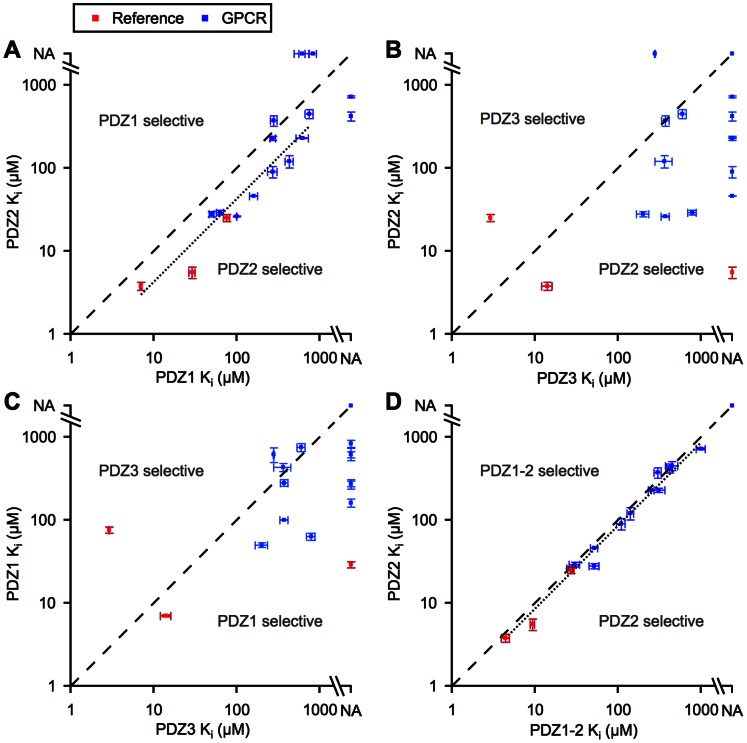
Domain preference and peptide selectivity. Comparison of *K*_i_ values for binding of GPCR and reference (KIF1Bα, GluN2B, and CRIPT) C-tail peptides to PSD-95 PDZ2 and PDZ1 (A), PDZ2 and PDZ3 (B), PDZ1 and PDZ3 (C), and isolated PDZ2 and PDZ2 in the PDZ1-2 supramodule (D). The dashed lines represent identical *K*_i_ values for the two domains. The dotted lines in A and D are line fits to the data points; C-tails binding to only one of the domains were omitted from the fitting. NA, no affinity, defined as a *K*_i_ value above 1000 µM. Error bars represent fitting errors or the standard error of the mean (see Table 1).

The *K*_i_ values obtained for each of the PSD-95 PDZ domains facilitate analysis of the PDZ domain preference of the receptors and the peptide-binding selectivity of the PDZ domains. We compared the binding to the different PDZ domains pairwise using scatter plots of the *K*_i_ for binding of the C-tail peptides to each of the two domains. If the C-tails bound to the domains with the same affinity, the points should be distributed around a line with a slope of 1 (the dashed line in Figure 3A–D). Comparison of the binding to PDZ1 and PDZ2 showed that most C-tail peptides bound preferentially to PDZ2 (Figure 3A). Three receptors (the metabotropic glutamate receptors mGlu_1(a)_ and mGlu_7(a)_ receptors and SSTR3) deviated from this trend by binding to PDZ1, but not to PDZ2; all three receptors were weaker binding receptors with *K*_i_ above 600 µM. Further analysis of the PDZ1/PDZ2 scatter plot showed that the points were distributed around a line that was parallel to the dashed line, but shifted 1.7–3.2 times (95% confidence interval) along the x-axis. This suggests that the peptide-binding selectivity of PDZ1 and PDZ2 is the same, but the *K*_i_ for binding to PDZ2 is 1.7–3.2 times lower than for binding to PDZ1.

Comparison of PDZ3 with PDZ1 and PDZ2 showed that most C-tail peptides bound preferentially to PDZ1 and PDZ2 over PDZ3, but, in contrast to the PDZ1/PDZ2 scatter plot, there was no trend in the distribution of the points (Figure 3B–C). The only PDZ3 selective C-tail, besides the reference peptide CRIPT, was SSTR3. This shows that the peptide-binding selectivity of PDZ3 is different from the selectivity of PDZ1 and PDZ2 and that PDZ3 selective receptors are less abundant than PDZ1 and PDZ2 selective receptors in our C-tail peptide library.

### PDZ1-2 Supramodule

The first two PDZ domains of PSD-95 constitute a supramodule, PDZ1-2, which has been hypothesized to have binding properties that are different from those of the isolated domains [8]. Using the same approach as for the isolated domains, we compared the *K*_i_ for binding of C-tail peptides to the PDZ2 part of the PDZ1-2 supramodule (PDZ2*) with binding to isolated PDZ2 under the assumption that the PDZ2 selective probe Cy5-GluN2B is also selective for PDZ2*. Comparison of binding to isolated PDZ2 and binding to PDZ2* showed that the points were all positioned close to the dashed line (Figure 3D), indicating that the binding characteristics of isolated PDZ2 and PDZ2* were similar. Closer analysis showed that the *K*_i_ for binding to PDZ2* was 1.04–1.32 (95% confidence interval) times higher than the *K*_i_ for binding to isolated PDZ2. Although significant, this minor difference should not change the conclusions obtained with the isolated PDZ2 domain. In conclusion, the peptide-binding selectivity is the same for isolated PDZ2 and PDZ2* and the affinity is similar, which is consistent with a previous study of the GluN2B C-tail binding to PDZ2* [25].

### Colocalization of Proteins in Cells

We used confocal fluorescence microscopy to study the colocalization and potential for mutual regulation of full-length GPCRs and PSD-95 in a complex cellular environment, and compare to the *in vitro* observations.

Cells were cotransfected with plasmids encoding GFP-tagged PSD-95 (PSD-95-GFP) and GPCRs fused to SNAP-tag (SNAP-GPCR) [26]. The receptors on the cell surface were fluorescently labeled by an irreversible reaction between the SNAP-tag and the cell-impermeable substrate BG-647, thus eliminating the background signal from intracellular receptor populations. We used line scans across the membrane to evaluate the distribution of PSD-95-GFP and SNAP-GPCRs near the plasma membrane.

**Figure 4:**
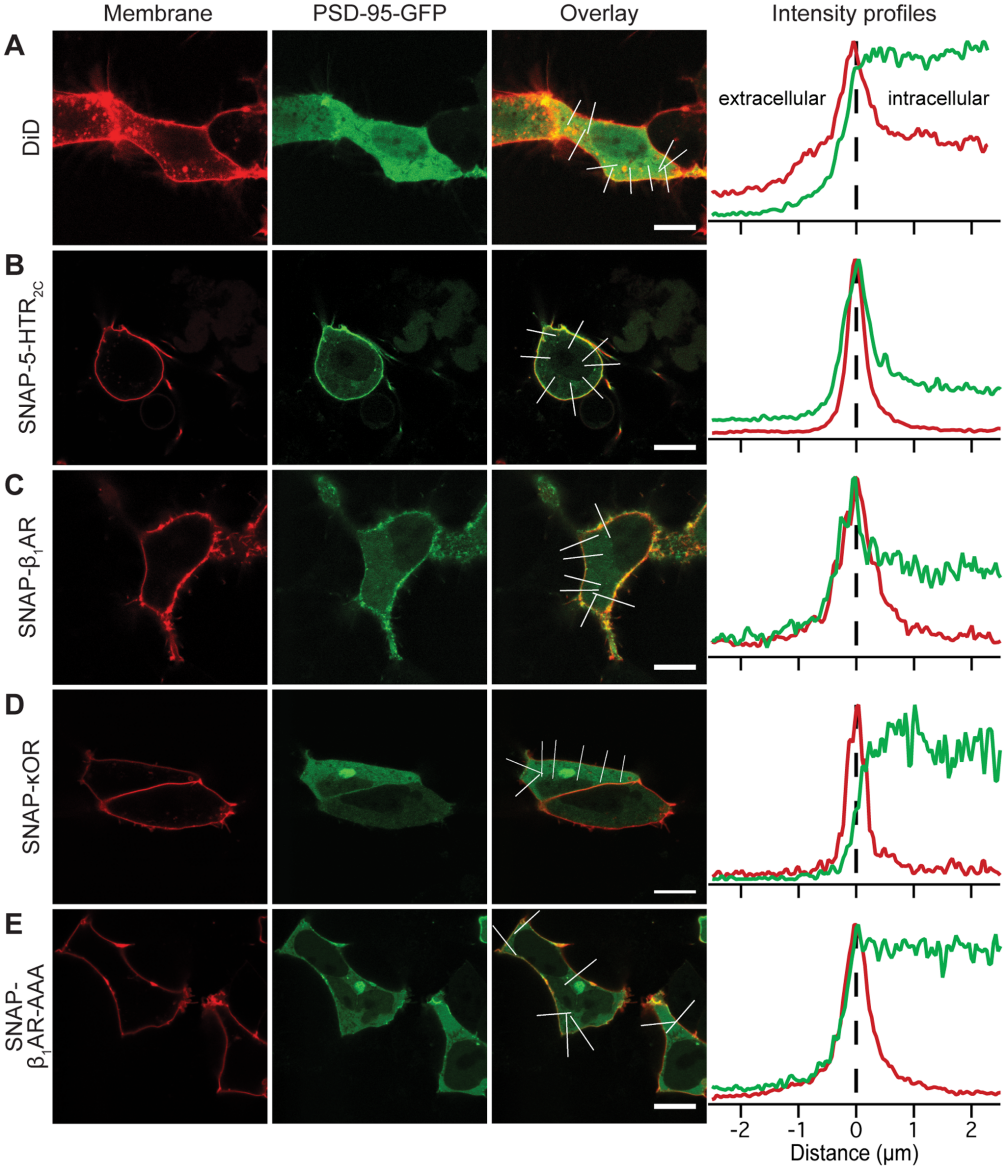
Validation of colocalization assay. PSD-95 is visualized by fusion to GFP, the receptors by fusion to SNAP-tag and labeling with the fluorescent SNAP-tag substrate BG-647. Cells transfected only with PSD-95-GFP were stained with DiD to visualize the plasma membrane. (A) Cells transfected with PSD-95-GFP only. (B–F) Cells coexpressing PSD-95-GFP and SNAP-5-HTR_2C_ (B), SNAP-β_1_AR (C), SNAP-κOR (D), or SNAP-β_1_AR-AAA (E). The graphs (right) show averaged line scans along the regions of interest indicated on the overlay images. The signal from SNAP-GPCRs and the membrane dye is shown in red; PSD-95-GFP is shown in green. Scale bars are 10 µm.

As a reference, the distribution of PSD-95-GFP in the absence of overexpressed GPCR was analyzed in cells labeled with the membrane dye DiD. Under those conditions, PSD-95-GFP was distributed evenly in the cytosol, as illustrated on the confocal micrographs and in the line profiles (Figure 4A). When cells coexpressed SNAP-5-HTR_2C_ or SNAP-β_1_AR and PSD-95-GFP, PSD-95-GFP was partly redistributed to the cell membrane (Figure 4B–C). The line profiles show that the PSD-95-GFP signal peaks at the membrane and then decreases steeply to a constant lower level in the cytosol (Figure 4B–C, right panel).

In contrast, the PSD-95-GFP distribution in cells coexpressing a receptor without PDZ motif, SNAP-κ opioid receptor (κOR, RDIDGMNKPV–COO^–^), or a receptor where the PDZ motif is disrupted by adding three alanines to the C-terminus of the receptor, SNAP-β_1_AR-AAA, was similar to the distribution in cells without overexpressed receptor (Figure 4D–E). This indicates that the observed redistributions indeed result from specific PDZ interactions. These results are consistent with the *in vitro* results, showing PSD-95 interactions with 5-HTR_2C_ and β_1_AR and no interaction with κOR, and with the literature [11], [13], [15].

**Figure 5:**
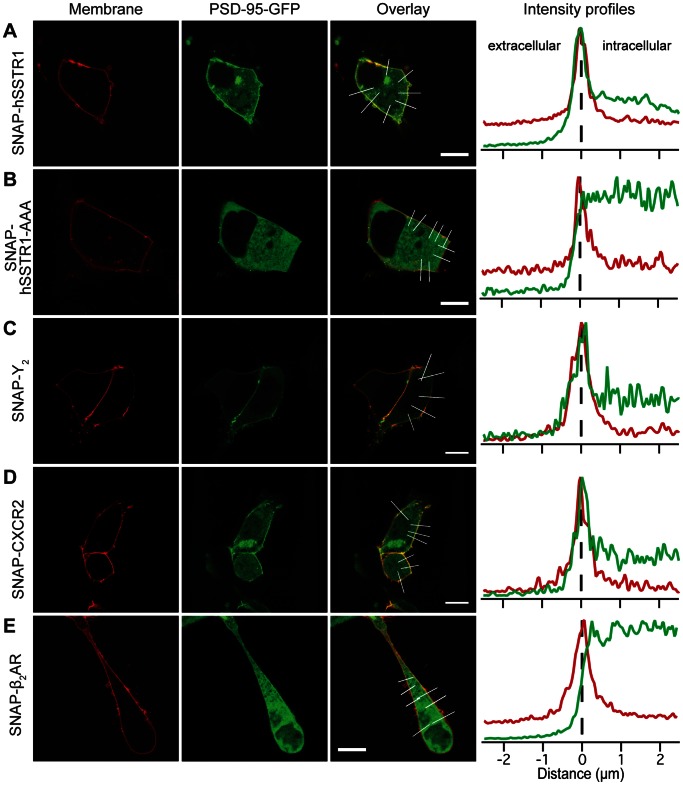
GPCR–PSD-95 interactions identified by FP colocalize in cells. PSD-95 is visualized by fusion to GFP, the receptors by fusion to SNAP-tag and labeling with BG-647. Cells coexpressing PSD-95-GFP and SNAP-hSSTR1 (A), SNAP-hSSTR1-AAA (B), SNAP-Y_2_ (C), SNAP-CXCR2 (D), or SNAP-β_2_AR (E). The graphs (right) show averaged line scans along the regions of interest indicated on the overlay images. The signal from SNAP-GPCRs is shown in red; PSD-95-GFP is shown in green. Scale bars are 10 µm.

We then looked at the strongest binder among the identified *in vitro* interactions, hSSTR1: Coexpression of PSD-95-GFP and SNAP-hSSTR1 resulted in a distribution pattern similar to the distribution in cells cotransfected with SNAP-5-HTR_2C_ (Figure 5A). Moreover, when the hSSTR1 PDZ motif was disrupted by adding three alanines to the C-terminus of the receptor (SNAP-hSSTR1-AAA), no redistribution of PSD-95-GFP was observed (Figure 5B). Together, these results indicate that the colocalization of hSSTR1 and PSD-95 is governed by a specific, PDZ domain-mediated interaction.

Similarly, we observed redistribution of PSD-95-GFP to the membrane upon coexpression with SNAP-Y_2_ or SNAP-CXCR2 (Figure 5C–D), indicating that these receptors associated with PSD-95 in cells. As for the interaction with mSSTR4 identified in the literature, we measured the *in vitro K*_i_ values by FP for the interactions between the PSD-95 PDZ domains and CXCR2 and the Y_2_ receptor to be in the 200–450 µM range (the observed *K*_d_ in cells is most likely much lower, which is discussed in detail later). These interactions are weaker *in vitro* than most of the interactions identified in the literature, suggesting that there is a potential for discovery of many new GPCR–PDZ interactions within this range.

The interaction between β_2_AR and PSD-95 was among the weakest we quantified by FP (*K*_i_ = 720 µM). Consistent with this, we could not detect a redistribution of PSD-95-GFP upon coexpression with SNAP-β_2_AR (Figure 5E). This interaction is thus possibly too weak to be detected by our approach.

The colocalization between PSD-95 and the GPCRs tested here is in excellent agreement with our *in vitro* results. Furthermore, we show that the colocalizations between PSD-95 and hSSTR1 and β_1_AR are mediated by a PDZ motif. This indicates that the colocalizations are in fact mediated by direct interactions between the PDZ domains of PSD-95 and the GPCR C-tails. It should, however, be kept in mind that our assay is not able to distinguish the presence of additional proteins involved in the interaction between PSD-95 and the GPCR C-tail in a cellular environment.

### Endogenous mSSTR1 Colocalizes with PSD-95 in Primary Neurons

**Figure 6:**
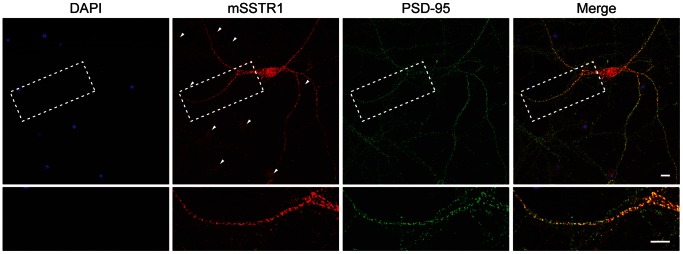
mSSTR1 and PSD-95 colocalize in primary hippocampal neurons. Mouse neurons cultivated for 20 days *in vitro* were stained for mSSTR1 (red signal) and PSD-95 (green signal). Yellow color in the merged picture (right panels) indicates colocalization of mSSTR1 and PSD-95 and is particularly seen on dendritic spines. The lower panels show a magnification of the dashed region depicted in the overviews. Nuclei of primary neurons were counterstained with DAPI (left panels). Several neurons were not stained by the anti-SSTR1 antibody (indicated by arrowheads), indicating that the mSSTR1 staining is specific. Scale bars are 10 µm.

To test whether endogenous mSSTR1 and PSD-95 are naturally expressed in the same cells and colocalize at physiolocal concentrations of the proteins, we analyzed the cellular localization of endogenous mSSTR1 and PSD-95 in cultured primary murine hippocampal neurons using immunocytochemistry with antibodies interacting specifically with mSSTR1 (Figure 6) and PSD-95 [27] (Figure S2). mSSTR1 was in many neurons detected in dendrites and at the soma in fluorescent clusters. Costaining with anti-PSD-95 clearly indicated a colocalization of both proteins, most likely at postsynaptic sites on dendritic spines (Figure 6). These data indicate that neuronal mSSTR1 is spatially associated with PSD-95 in cultured neurons, which is a prerequisite for physical interaction and thus supports further investigation of the physiological relevance of the identified mSSTR1–PSD-95 interaction.

## Discussion

We here present the first quantitative characterization of the kinetics and affinity of interactions between GPCRs and one of the best characterized PDZ scaffold proteins, PSD-95, using a generic approach that is straightforward to expand to other scaffold proteins. We determined the *K*_d_ of interactions between peptides mimicking GPCR C-tails and the isolated PDZ domains of PSD-95 by FP. The *K*_d_ values were confirmed by SPR and we could furthermore estimate the upper limit for the residence time of GPCR–PSD-95 interactions. These *in vitro* findings were consistent with the co-localization of full-length GPCRs and PSD-95 in HEK293 cells and finally, we found that SSTR1 colocalized with PSD-95 in mouse hippocampal neurons.

### Predicting GPCR–PDZ Association in Cells Based on *in vitro* Affinities

We measured the affinity of the PSD-95 PDZ domains for 15 GPCR C-tails. Even though we find a good qualitative correlation between *in vitro* and cell data, the *in vitro* affinities are not likely to correspond to the apparent affinities in cells. They are, however, still very useful and can be used to determine which interactions are most likely to occur in a cellular environment, as we have shown here. Moreover, GPCRs can be ranked on the basis of their affinities, which can be used to predict the outcome if several receptors interacting with the same PDZ domain are expressed in the same cell.

Apparent affinities in cells might differ from *in vitro* affinities for a number of reasons: First, PSD-95 has several interaction domains and is thus capable of binding more than one membrane embedded receptor at the same time, which can increase the apparent affinity, or avidity, by several orders of magnitude [19]; indeed, forming several weak transient interactions is a common feature of scaffold proteins [28]. Most of the receptors that interact with PSD-95 have a significant affinity for at least two of the PDZ domains and in some cases, e.g. Y_2_ and CXCR2, the affinity for two or three domains is practically identical, making this scenario very likely to occur. Second, PSD-95 is palmitoylated, which most likely positions it at the cell membrane in close proximity to the receptors, thus increasing the association rate [29]. Third, the environment in the cell is different from typical *in vitro* conditions: The presence of the plasma membrane, macromolecular crowding, and different electrolyte concentrations can influence the interaction. In fact, a high chloride concentration has been shown to decrease the affinity of PDZ motif peptides binding to PSD-95 PDZ2 and PDZ3 [24], [30], [31]. Fourth, the affinity can be modulated either allosterically by interaction with another protein or by posttranslational modification of either the GPCR or PSD-95.

Based on the simplified assumption that the *K*_i_ for the strongest *in vitro* interaction with the PSD-95 PDZ domains is proportional to the strength of the interaction between the full-length proteins in cells, the GPCRs were divided into three groups: Group 1: *K*_i_ around 100 µM or less, approximately corresponding to the cut-off used in a large scale screening of PDZ interactions by a combination of protein microarray and FP [17], Group 2: *K*_i_ in the range 200–450 µM, and Group 3: *K*_i_ higher than 450 µM. Most of the PSD-95 interacting receptors identified in the literature belonged to Group 1 (5-HTR_2A_, 5-HTR_2C_, β_1_AR, BAI1 receptor, mSSTR1 (Table 1)). Interestingly, we found that the Group 2 receptors CXCR2 and the Y_2_ receptor colocalized with full-length PSD-95 in cells. Furthermore, Group 2 contains SSTR4, which has previously been shown to co-immunoprecipitate with PSD-95 from rat brain (the C-tail of rat and mouse SSTR4 is identical) [14] and together, these results indicate that receptors in Group 2 are good candidates for physiologically relevant interactions. This is a significant expansion of the range of interactions that is usually investigated further, which could lead to the discovery of many new GPCR–PDZ interactions. Indeed, the physiologically relevant interaction between the cystic fibrosis transmembrane conductance regulator (CFTR) and the PDZ domain of the CFTR-associated ligand (CAL) has been shown to have a *K*_d_ above 600 µM *in vitro* [32].

### Kinetics of PDZ Domain Interactions: Implications for Screening Assays

We measured the binding of at least 5 GPCR C-tail peptides to each of the PSD-95 PDZ domains by time resolved SPR and found that all of the C-tails dissociated with residence times below one second (τ <1 s). This is in agreement with values of τ = 0.014–0.22 s (*k*_d_ = 4.6–72 s^−1^) reported for stopped-flow fluorimetry measurements of PDZ domain interactions with known (non-GPCR) ligands [24]. The transient nature of GPCR–PDZ interactions is consistent with a role in organization of signaling complexes: it allows quick formation and dissociation of individual PDZ interactions in response to cellular events, while the modular design of PDZ scaffolds imparts a residence time of the full-length protein that is much longer than for the isolated domains and this stability is important for the function of PDZ scaffolds as organizational hubs.

Kinetics of PDZ domain interactions is also interesting from a methodological perspective, because most of the assays used to discover and characterize PDZ domain interactions require physical separation of the bound and free fractions, which is often done by immobilization on a solid support followed by a series of washing steps. Examples of such assays include affinity purification [13], [14], co-immunoprecipitation [10], [13], pull-down [2], and protein arrays [12], [33]. In those cases, the separation step can influence the outcome of the assay if a substantial fraction of complexes dissociate during the separation, which is often the case for transient interactions. This leads to underestimation of the affinity and a higher probability of false negatives and is most likely the reason why so few of the Group 2 interactions that we report here were discovered previously, whereas only one of the Group 1 interactions was new. Consequently, it is important to use separation independent methods, such as FRET, FP, and SPR, to characterize PDZ domain interactions.

### Interactions Identified or Confirmed

Two different isoforms of SSTR1 were used in this study: We showed by FP and SPR that human SSTR1 interacts with PSD-95 and showed colocalization of the proteins in HEK293 cells by confocal fluorescence microscopy. For mouse SSTR1, we showed by FP that it also interacts with PSD-95 *in vitro*, thus confirming a previous study [14] and in good agreement with a study on the interaction between mSSTR1 and a PSD-95 homolog, synapse-associated protein 97 [34]. We further demonstrated that mSSTR1 is coexpressed with PSD-95 in hippocampal neurons and is colocalized with PSD-95 at dendritic spines, which has not previously been demonstrated. Finally, the FP assay showed that both isoforms interact preferentially with the same domains of PSD-95, but the interaction with hSSTR1 was significantly stronger (P = 0.047, two-tailed, unpaired Student’s t test), probably due to slight differences in the −2 and the −6 positions of the two C-tail sequences (using the numbering scheme of Doyle *et al.* [35]). The clear colocalization of PSD-95 and the weaker binding mouse isoform of SSTR1 in hippocampal neurons suggests that hSSTR1 is also found in a complex with PSD-95 at physiological concentrations and in their native environment.

Besides SSTR1, we identified interactions of CXCR2 and the Y_2_ receptor with PSD-95 *in vitro* and observed colocalization of the proteins in HEK293 cells. Furthermore, we identified PSD-95 interactions with SSTR2A, SSTR3, hSSTR4, the mGlu_1(a)_ receptor, and the mGlu_7(a)_ receptor *in vitro*.

The interaction between β_2_AR and PSD-95 has been subject of some controversy: Joiner *et al.* have shown that the complete C-tail of β_2_AR binds PSD-95 PDZ3 *in vitro*, and that β_2_AR co-immunoprecipitates with PSD-95 from rat brain extract [16]. However, Hu *et al.* reported that β_2_AR does not interact with PSD-95 *in vitro* or in HEK293 cells [11]. In our hands, the interaction was close to the detection limit in the *in vitro* assays and we could not detect association in cells. Our results are thus most consistent with the results from Hu *et al.*

### Peptide-binding Selectivity

We found that the peptide-binding selectivity of PDZ1 and PDZ2 was similar, but PDZ2 generally bound peptides with higher affinity than PDZ1. This tendency was previously observed with the C-tail from nine different proteins in a general screening [17] and seen consistently during systematic investigations of the GluN2B C-tail [18], [36]. Two residues that participate in the stabilization of the C-terminal carboxylate group in the ligand are different in PDZ1 and PDZ2, namely Arg-70 and Gly-141 in PDZ1, which are Lys-165 and Tyr-236, respectively, in PDZ2 (Figure S3). These differences might explain why PDZ2 binds ligands with higher affinity than PDZ1, but the same selectivity.

PDZ3 showed a different peptide-binding selectivity than PDZ1 and PDZ2, but only few PDZ3 selective ligands were found. The difference in selectivity is probably partly due to differences in a number of the residues that contact the ligand side chains, for example residues 2 and 4 in β-strand βB and residues 4 and 5 in βC, and partly due to a 6 residue insert in the βB/βC loop (Figure S3). The lack of PDZ3 selective ligands has also been observed in screenings of broader ranges of natural ligands [17], [37], but a screening of artificial ligands showed that PDZ3 is as promiscuous as PDZ2 [37], thus suggesting that PDZ3 selective ligands are simply underrepresented in the genome.

### Conclusions

We have analyzed the interactions between a prototypical PDZ scaffold protein, PSD-95, and a wide range of GPCRs *in vitro* and in cells. We found by SPR that GPCR–PSD-95 PDZ interactions are transient (τ <1 s), which is consistent with a role in dynamic signaling events.

*In vitro* results obtained with FP and SPR showed that most of the GPCR–PSD-95 interactions identified in the literature have *K*_i_ values around 100 µM or lower (Group 1). The interaction between PSD-95 and SSTR1 was also in this range and we furthermore demonstrated that the endogenous full-length proteins colocalize in mouse hippocampal neurons. This shows that a curated, quantitative data set is useful for predicting which proteins that are likely to be associated in their native environment.

We discovered several new GPCR–PSD-95 interactions with *in vitro K*_i_ in the 200–450 µM range (Group 2), including CXCR2, the Y_2_ receptor, SSTR2A, SSTR3 and hSSTR4. Measurements in HEK293 cells showed that full-length CXCR2 and Y_2_ receptor also colocalize with PSD-95, suggesting that interactions in this range could be of physiological relevance. This could lead to the discovery of many new GPCR–PDZ interactions, as this affinity range has often been disregarded.

Our approach can easily be applied to other PDZ scaffolds with minimal changes in the experimental setup and even to other protein interaction domains, if the appropriate peptide library is established.

## Materials and Methods

### Expression and Purification of PSD-95 Constructs

Expression plasmids of PSD-95 PDZ1 (aa 61–151), PDZ2 (aa 155–249), PDZ3 (aa 309–401), and PDZ1-2 (aa 61–249) were constructed as described previously [36]. All PDZ constructs contained an additional N-terminal MHHHHHPRGS sequence for use in His-tag purification.

Chemically competent *E. coli* One Shot BL21 Star (DE3) bacteria were transformed with PDZ expressing plasmids. Protein expression was induced by addition of 0.5 mM isopropyl β-d-1-thiogalactopyranoside to bacterial culture at OD_600_ = 0.8–1.0, followed by 4 h growth at 37°C (PDZ1, PDZ2, or PDZ3), or at OD_600_ = 0.4–0.5, followed by overnight growth at 30°C (PDZ1-2).

Proteins were purified by immobilized metal ion affinity chromatography followed by size exclusion chromatography on an ÄKTApurifier 10 (GE Healthcare). Cell pellets with expressed protein were resuspended in buffer containing 4 mM sodium phosphate pH 7.4, 0.1 M NaCl, 20 mM imidazole, 1 mg/ml lysozyme, 1 mM PMSF, and EDTA-free Complete protease inhibitor cocktail (Roche) and incubated on ice for 30 min. While still on ice, the bacteria were lysed using a microtip sonicator in six cycles of 10 s high intensity bursts with 10 s pauses between bursts. Cell debris was pelleted by centrifugation at 48000×*g* for 45 min at 4°C followed by addition of 40 µg/ml DNase I and 10 mM MgCl_2_ to the lysate. After 15 min incubation on ice, the solution was cleared by centrifugation (48000×*g*, 10 min, 4°C) and filtration (0.45 µm filter) and finally diluted 3–4 times in Buffer A (20 mM sodium phosphate pH 7.4, 0.5 M NaCl) with 20 mM imidazole and loaded on a HisTrap HP 5 ml column (GE Healthcare) equilibrated in Buffer A with 20 mM imidazole. Each PDZ protein was eluted with Buffer A with 250 mM imidazole, and fractions containing protein were pooled, concentrated with Amicon Ultra-15 MWCO 3000 (PDZ1, -2, -3) or 10000 (PDZ1-2) centrifugal filter devices and further purified on a Superdex 75 10/300 GL column equilibrated in a buffer containing 10 mM HEPES pH 7.4 and 150 mM NaCl. Again, protein containing fractions were pooled and concentrated with Amicon centrifugal filter devices and finally frozen in liquid nitrogen and stored at −80°C. Size and purity (>95%) of the proteins were checked by SDS-PAGE stained with SimplyBlue SafeStain (Invitrogen). Protein concentrations were determined from the absorbance at 280 nm, using molar extinction coefficients determined by amino acid analysis (Alphalyse, Odense, Denmark).

### Synthesis of C-tail Peptides

Cy5-labeled NMDA receptor GluN2B subunit, CRIPT, and KIF1Bα peptides and unlabeled GluN2B and CRIPT peptides were synthesized and labeled as previously described [36]. The remaining peptides were synthesized by Schafer-N (Copenhagen, Denmark). Unlabeled KIF1Bα peptide and the GPCR peptides corresponded to the 10 C-terminal aa of the proteins with an N-terminal YDDR/DDR linker to increase solubility and enable spectrophotometrical determination of the concentration (Tyr was omitted for peptides already containing Tyr or Trp). Concentrations of unlabeled peptides were determined from the absorbance at 280 or 293 nm by diluting the peptide stock in 0.1 M NaOH, and the concentrations of Cy5-labeled peptides were determined from the absorbance at 650 nm.

### Fluorescence Polarization Assay

FP measurements were performed in black, flat bottom 384-well NBS microplates (Corning) on a Synergy H4 microplatereader (BioTek Instruments) equipped with a 620/40 nm excitation filter, a 680/30 nm emission filter, and a 660 nm dichroic mirror. Polarization values were corrected with blank samples and samples with fluorescent probe alone. At least 10 data points were measured for each curve, and each data point was measured in triplicate. 25 nM Cy5-labeled probe was used for each assay: Cy5-KIF1Bα for PDZ1, Cy5-GluN2B for PDZ2 and PDZ1-2, and Cy5-CRIPT for PDZ3. All FP measurements were performed in a buffer containing 10 mM HEPES pH 7.4, 150 mM NaCl, and 1% BSA and incubated at least 15 min at room temperature before reading. The signal was stable for more than an hour, showing that the reaction was at equilibrium.

Saturation binding curves were used to determine the functionality of the PDZ domains by comparison with literature values and to calculate *K*_i_ values from competition curves. Saturation binding curves were performed by mixing probe with a twofold dilution series of PDZ protein in a total volume of 30 µl. The *K*_d_ was determined by fitting to a 1∶1 binding model: Y = Y_max_ × X/(X+*K*_d_).

For the competition binding curves, probe was mixed with a constant concentration of PDZ protein corresponding to the *K*_d_ and a twofold dilution series of unlabeled peptide in a total volume of 30 µl. The baseline was determined using a sample containing only probe. The IC_50_ was determined by fitting to a 1∶1 binding model: Y = Y_max_ − Y_max_ × X/(X+IC_50_). The *K*_i_ was calculated from the IC_50_ and from the *K*_d_ for the interaction between the PDZ protein and the probe as described previously [38].

### Surface Plasmon Resonance Assay

A Biacore X100 (GE Healthcare) SPR instrument equilibrated to 25.0°C and equipped with a Sensor Chip CM5 (for PDZ1 and PDZ2) or CM4 (for PDZ3) (GE Healthcare) was used for the SPR measurements. The running buffer was HBS-EP+ (10 mM HEPES pH 7.4, 150 mM NaCl, 3 mM EDTA, 0.05% Surfactant P-20 (GE Healthcare)).

PDZ domains were immobilized by amine coupling at a flow rate of 5 µl/min on a surface activated by a 7 min injection of a 1∶1 mixture of 0.4 M 1-ethyl-3-(3-dimethylaminopropyl) carbodiimide (EDC) and 0.1 M N-hydroxysuccinimide (NHS). The remaining active groups were deactivated by a 7 min injection of 1 M ethanolamine HCl pH 8.5. 750 RU PDZ1, 780 RU PDZ2, and 410 RU PDZ3 were immobilized.

GPCR C-tail peptide samples were injected in increasing concentrations for 1 min with 1 min dissociation time at 30 µl/min. Reproducibility was tested by injecting the 20 µM sample again after the highest concentration sample. The binding curves were corrected by subtraction of buffer blanks and the response from a reference surface that was activated with EDC and NHS and deactivated with ethanolamine like the surface with immobilized PDZ domain. For peptides with a cysteine, 1 mM dithiothreitol (DTT) was added to the samples. Steady-state response curves were plotted from the responses 10 s before injection end. The *K*_d_ was determined by fitting steady-state response curves to a 1∶1 binding model with a linear component to corrrect for low affinity binding, probably resulting from the immobilization: Y = Y_max_ × X/(X+*K*_d_)+B × X.

*K*_i_ scatter plots were fitted by linear models after log-transforming the data. If the fitted slope was close to unity (within the standard deviation), it was fixed to 1. 95% confidence intervals were calculated from the fitting errors.

### Colocalization in HEK293 Cells

HEK293 cells were cultured at 37°C in DMEM supplemented with 2.2% FBS, in a 5% CO_2_ atmosphere with 95% humidity. Cells were plated on sterile glass cover slips two days before an experiment. Following overnight incubation, adherent cells were transiently transfected with the appropriate plasmids in a 1∶1 ratio, using TurboFect (Fermentas) as transfection reagent, according to the protocol from the manufacturer. Finally, cells were cultured overnight for protein expression.

PSD-95-GFP was kindly provided by Philippe Marin (Institut de Génomique Fonctionnelle, Montpellier, France) [39]. Plasmids for SNAP-tagged receptors (5-HTR_2C_, β_1_AR, β_2_AR, κOR, and hSSTR1) were obtained from Cisbio (France). For SNAP-hSSTR1-AAA and SNAP-β_1_AR-AAA, three alanine residues were added to the C-terminus of the respective wild type plasmids (GenScript, Piscataway, NJ, USA).

SNAP-GPCR constructs were fluorescently labeled with fluorescent *O* ^6^-benzylguanine (BG)-647 (New England Biolabs), according to the protocol from the manufacturer. In short, cells were incubated for 10 min with 5 µM BG-647 at 37°C and then washed. In samples that were not transfected with a receptor, cell membranes were stained with Vybrant DiD cell-labeling solution (Invitrogen). Subsequently, the cells were imaged in serum-free DMEM with HEPES (Invitrogen).

Cross section micrographs of cells were acquired on an inverted confocal microscope (TCS SP5, Leica), using a water immersion objective (63x magnification, numerical aperture 1.2). GFP was excited with a wavelength of 488 nm; BG-647 and DiD were excited with a wavelength of 633 nm.

Images were digitally processed with ImageJ [40] to quantify the recruitment of PSD-95-GFP to the membrane and the colocalization with GPCRs. Straight line regions of interest perpendicular to the plasma membrane and covering several micrometers inside and outside the cell were selected manually, and the fluorescence intensities along these line segments were measured for both the receptor and PSD-95-GFP. These signals were further treated using Igor Pro (WaveMetrics). In brief, the position of the membrane was determined from the receptor fluorescence and set to 0 µm. At least five traces from the same cell were averaged and normalized for each graph.

### Culture of Primary Hippocampal Neurons and Immunocytochemistry

Primary hippocampal neuronal cell cultures were prepared from 18.5 days *post coitum* mouse embryos as previously described [41]. Pregnant mothers were sacrificed by an overdose of isoflurane and embryos by decapitation approved by the Guide for the Care and Use of Laboratory Animals of the Government of Upper Bavaria (Germany) as well as by the Animal Care and Use Committee of the Max Planck Institute of Psychiatry (Munich, Germany). Dissociated neurons were grown in Neurobasal-A medium supplemented with B27 Supplement (Invitrogen) and GlutaMAXI (Invitrogen). Neurons were plated on coverslips (Menzel) coated with 50 µg/ml poly-d-lysine (Sigma) and 5 µg/ml laminin (Invitrogen). After 20 days *in vitro*, neurons were fixed with 4% paraformaldehyde containing 4% sucrose. Immunohistochemistry was carried out as described [42], using the following antibodies: anti-SSTR1 (1∶50; Novus Biologicals, NB120-2366), anti-PSD-95 (1∶500; Neuromab, 75-028), anti-rabbit conjugated to Alexa-Fluor 594 (1∶1000; Invitrogen, A11037) and anti-mouse conjugated to Alexa-Fluor 488 (1∶1000; Invitrogen, A11029). To confirm the specificity of the antibodies used to detect SSTR1 and PSD-95, immunohistochemistry was performed as above but omitting the first antibody (Figure S2). Nuclei were counterstained using DAPI. Immunocytochemical analysis was carried out by laser-scanning confocal microscopy. Images were acquired simultaneously in two acquisition channels with the FLUOVIEW FV 1000 (version 2.0a) acquisition analyzer program. Images were digitalized using Image J, Adobe Photoshop CS2, and Adobe Illustrator CS2.

## Supporting Information

Figure S1 **Correlation between SPR and FP binding data.** Scatter plot of *K*_i_ values for GPCR C-tails binding to each of the PSD-95 PDZ domains measured by FP versus *K*_d_ values for the same interactions measured by SPR. The dashed line indicates a perfect correlation between the data. The dotted line is a line fit (Y = A+B × X) to log-transformed data and it shows that the SPR data on average gives *K*_d_ values that are 2 times higher than the *K*_i_ values found by FP.
(TIF)

Figure S2 **Assessement of the specificity of antibodies against mSSTR1 and PSD-95 in primary hippocampal neurons.** Mouse neurons cultivated for 20 days *in vitro* were stained for mSSTR1 (red signal, left panel) and PSD-95 (green signal, right panel) using only secondary antibodies. Nuclei of primary neurons were counterstained with 4′,6-diamidino-2-phenylindole (DAPI). No nonspecific staining was detectable. Scale bars = 10 µm.
(TIF)

Figure S3 **Sequence alignment of PSD-95 PDZ1, PDZ2, and PDZ3.** Residues that are predicted to contact the ligand are highlighted in gray [35], [43]. Secondary structure elements are indicated above the sequence [35], notice that helix αC is only found in PDZ3. Identical residues are indicated with asterisks; conserved and semi-conserved residues are indicated with colons and dots, respectively.
(TIF)

Table S1 Sequences of C-terminal tail peptides used for fluorescence polarization and surface plasmon resonance experiments.
(PDF)

Table S2 Comparison of *K*_i_ values for reference protein interactions with *K*_i_ and *K*_d_ values from the literature.
(PDF)

Table S3 GPCRs found not to interact with PSD-95 using fluorescence polarization.
(PDF)

Table S4 *K*_d_ values for interactions with the PSD-95 PDZ domains determined by surface plasmon resonance.
(PDF)
