# Supplementary figures and images for: PDZ Domain-Mediated Interactions of G Protein-Coupled Receptors with Postsynaptic Density Protein 95: Quantitative Characterization of Interactions
Source: PLoS One. 2013 May 14;8(5):e63352. doi: 10.1371/journal.pone.0063352 (PMC3653948; doi:10.1371/journal.pone.0063352)

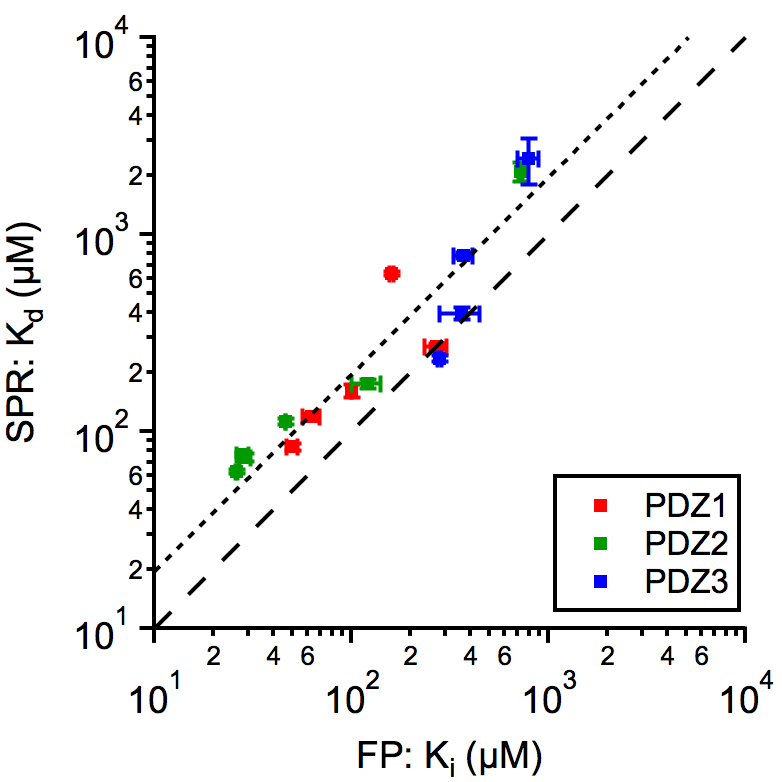

Supplement: Figure S1 — Correlation between SPR and FP binding data. Scatter plot of K i values for GPCR C-tails binding to each of the PSD-95 PDZ domains measured by FP versus K d values for the same interactions measured by SPR. The dashed line indicates a perfect correlation between the data. The dotted line is a line fit (Y = A+B × X) to log-transformed data and it shows that the SPR data on average gives K d values that are 2 times higher than the K i values found by FP. (TIF) [file pone.0063352.s001.tif]

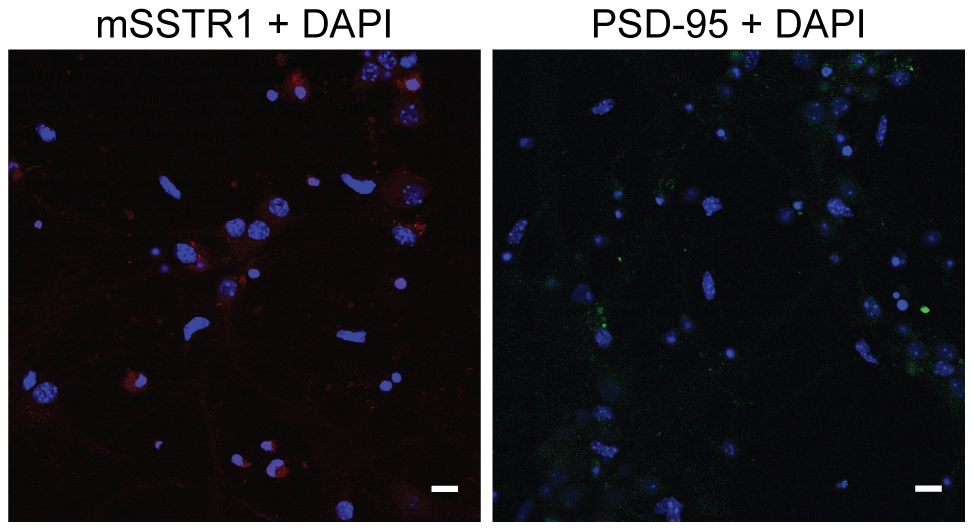

Supplement: Figure S2 — Assessement of the specificity of antibodies against mSSTR1 and PSD-95 in primary hippocampal neurons. Mouse neurons cultivated for 20 days in vitro were stained for mSSTR1 (red signal, left panel) and PSD-95 (green signal, right panel) using only secondary antibodies. Nuclei of primary neurons were counterstained with 4′,6-diamidino-2-phenylindole (DAPI). No nonspecific staining was detectable. Scale bars = 10 µm. (TIF) [file pone.0063352.s002.tif]

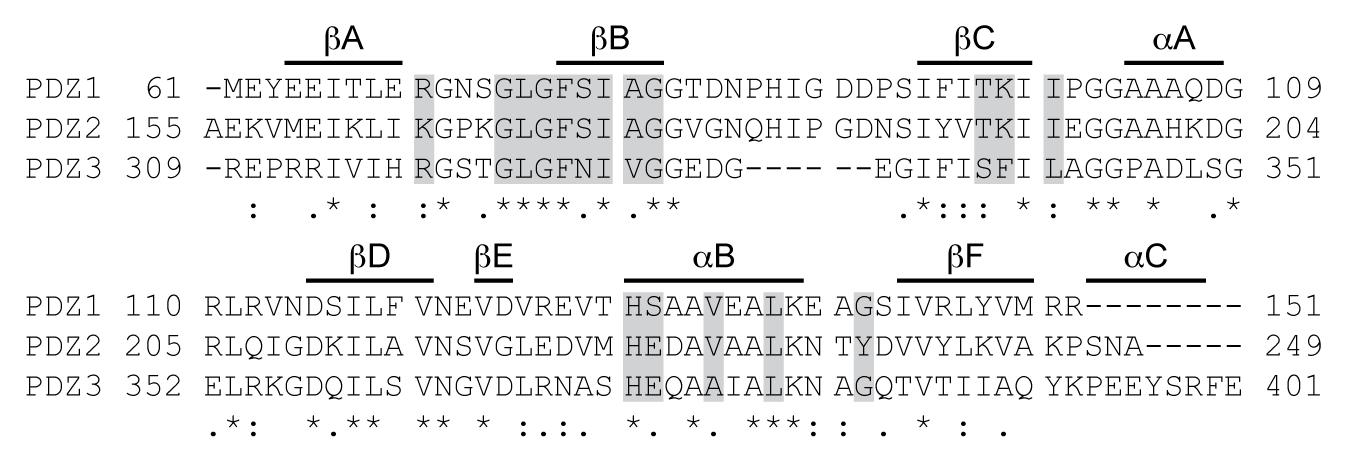

Supplement: Figure S3 — Sequence alignment of PSD-95 PDZ1, PDZ2, and PDZ3. Residues that are predicted to contact the ligand are highlighted in gray [35], [43]. Secondary structure elements are indicated above the sequence [35], notice that helix αC is only found in PDZ3. Identical residues are indicated with asterisks; conserved and semi-conserved residues are indicated with colons and dots, respectively. (TIF) [file pone.0063352.s003.tif]
