# Supplementary material for: PDZ Domain-Mediated Interactions of G Protein-Coupled Receptors with Postsynaptic Density Protein 95: Quantitative Characterization of Interactions
Source: PLoS One. 2013 May 14;8(5):e63352. doi: 10.1371/journal.pone.0063352 (PMC3653948; doi:10.1371/journal.pone.0063352)
Supplement: Table S1 — Sequences of C-terminal tail peptides used for fluorescence polarization and surface plasmon resonance experiments. (PDF) [file pone.0063352.s004.pdf]

Table S1. Sequences of C-terminal tail peptides used for fluorescence polarization and surface plasmon resonance experiments.

Protein	Species	Family	C-tail sequence	Class
**Reference proteins**				
CRIPT	Human	CRIPT	LDTKNYKQTSV	I
GluN2B	Human	Glutamate gated ion channel	YEKLSSIESDV	I
KIF1Bα	Human	Kinesin-like	NLKAGRETTV	I
**G protein-coupled receptors**				
5-HTR_{2A}	Human	5-hydroxytryptamine receptors	DGVNEKVSCV	I
5-HTR_{2C}	Human	5-hydroxytryptamine receptors	SVVSERISSV	I
5-$HTR_{4(a)}$	Human	5-hydroxytryptamine receptors	NDPESLESCF	I
A_{2B}	Human	Adenosine receptors	GVQPALGVGL	II
β_1AR	Human	Adrenergic receptors	RPGFASESKV	I
β_2AR	Human	Adrenergic receptors	RNCSTNDSLL	I
CCR5	Human	Chemokine receptors	TGEQEISVGL	II
CCR5	Mouse	Chemokine receptors	TGEHEVSTGL	I
CXCR1	Human	Chemokine receptors	SSSVNVSSNL	I
CXCR2	Human	Chemokine receptors	SSSGHTSTTL	I
CXCR3	Human	Chemokine receptors	ETSEASYSGL	I
CXCR4	Human	Chemokine receptors	ESESSSFHSS	
CXCR5	Human	Chemokine receptors	SENATSLTTF	I
BAI1	Human	Class B orphans	QDIIDLQTEV	I
Ghrelin	Human	Ghrelin receptor	RAWTESSINT	
LPA_2	Human	Lysophospholipid receptors	GHPLMDSTL	I
$mGlu_{1(a)}$	Human	Metabotropic glutamate receptors	RDYKQSSSTL	I
$mGlu_2$	Human	Metabotropic glutamate receptors	EVVDSTTSSL	I
$mGlu_{5(b)}$	Human	Metabotropic glutamate receptors	RDYTQSSSSL	I
$mGlu_{7(a)}$	Human	Metabotropic glutamate receptors	KYVSYNNLVI	II
Y_2	Human	Neuropeptide Y receptors	NDSFTEATNV	I
δOR	Human	Opioid receptors	SDGPGGGAAA	II
κOR	Human	Opioid receptors	RDIDGMNKPV	
μOR-1A	Human	Opioid receptors	DRTNHQVRSL	
μOR-1B2	Human	Opioid receptors	QRERRQKSDW	I
μOR-1B5	Human	Opioid receptors	QSPFPFPGRV	
$P2Y_1$	Human	P2Y receptors	EFKQNGDTSL	I
SSTR1	Human	Somatostatin receptors	GTCTSRITTL	I
SSTR1	Mouse	Somatostatin receptors	GTCASRISTL	I
SSTR2A	Human	Somatostatin receptors	LLNGDLQTSI	I
SSTR3	Human	Somatostatin receptors	KSSTMRISYL	I
SSTR4	Human	Somatostatin receptors	RIPLTRTTTF	I
SSTR4	Mouse	Somatostatin receptors	QVPFTKTTTF	I
SSTR5	Human	Somatostatin receptors	ANGLMQTSKL	I
