# Supplementary material for: PDZ Domain-Mediated Interactions of G Protein-Coupled Receptors with Postsynaptic Density Protein 95: Quantitative Characterization of Interactions
Source: PLoS One. 2013 May 14;8(5):e63352. doi: 10.1371/journal.pone.0063352 (PMC3653948; doi:10.1371/journal.pone.0063352)
Supplement: Table S2 — Comparison of K i values for reference protein interactions with K i and K d values from the literature. (PDF) [file pone.0063352.s005.pdf]

Table S2. Comparison of K_i values for reference protein interactions with K_i and K_d values from the literature.

			K_i (µM)[a,b]					
			**PDZ1**		**PDZ2**		**PDZ3**	
**Competitor**	**Species**	**Family**	**Literature**	**Result**	**Literature**	**Result**	**Literature**	**Result**
CRIPT	Human	CRIPT	97 ± 18[c,d]	76 ± 6.7	25 ± 1.6[c,d]	25 ± 2.5	2.1 ± 0.15[c,d]	2.9 ± 0.01[c]
GluN2B	Human	Glutamate gated ion channel	18 ± 0.92[c,d]	29 ± 2.5[c]	4.1 ± 0.17[c,d]	5.5 ± 0.89[c]	NA[d]	NA
KIF1Bα	Human	Kinesin-like	2.4[e,f]	7 ± 0.2	2.1[e,f]	3.8 ± 0.42	2.7[e,f]	14 ± 2.2

[a]The shown data are K_i ± fitting error, unless otherwise noted.

[b]NA, no affinity, defined as a K_i value above 1000 µM.

[c]Shown data are mean K_i ± standard error of the mean from two or more independent experiments.

[d]Bach A, Chi CN, Olsen TB, Pedersen SW, Røder MU, et al. (2008) Modified peptides as potent inhibitors of the postsynaptic density-95/*N*-methyl-D-aspartate receptor interaction. J Med Chem 51: 6450-6459.

[e]K_d value.

[f]Stiffler MA, Chen JR, Grantcharova VP, Lei Y, Fuchs D, et al. (2007) PDZ binding selectivity is optimized across the mouse proteome. Science 317: 364-369.
