# Supplementary material for: PDZ Domain-Mediated Interactions of G Protein-Coupled Receptors with Postsynaptic Density Protein 95: Quantitative Characterization of Interactions
Source: PLoS One. 2013 May 14;8(5):e63352. doi: 10.1371/journal.pone.0063352 (PMC3653948; doi:10.1371/journal.pone.0063352)
Supplement: Table S3 — GPCRs found not to interact with PSD-95 using fluorescence polarization. (PDF) [file pone.0063352.s006.pdf]

Table S3. GPCRs found not to interact with PSD-95 using fluorescence polarization.

**Competitor**	**Species**	**Family**
5-HTR$_{4(a)}$	Human	5-Hydroxytryptamine receptors
A$_{2B}$	Human	Adenosine receptors
CCR5	Human	Chemokine receptors
CCR5	Mouse	Chemokine receptors
CXCR1	Human	Chemokine receptors
CXCR3	Human	Chemokine receptors
CXCR4	Human	Chemokine receptors
CXCR5	Human	Chemokine receptors
Ghrelin	Human	Ghrelin receptor
LPA$_2$	Human	Lysophospholipid receptors
mGlu$_2$	Human	Metabotropic glutamate receptors
mGlu$_{5(b)}$	Human	Metabotropic glutamate receptors
δOR	Human	Opioid receptors
κOR	Human	Opioid receptors
μOR-1A	Human	Opioid receptors
μOR-1B2	Human	Opioid receptors
μOR-1B5	Human	Opioid receptors
P2Y$_1$	Human	P2Y receptors
SSTR5	Human	Somatostatin receptors
