# Supplementary material for: PDZ Domain-Mediated Interactions of G Protein-Coupled Receptors with Postsynaptic Density Protein 95: Quantitative Characterization of Interactions
Source: PLoS One. 2013 May 14;8(5):e63352. doi: 10.1371/journal.pone.0063352 (PMC3653948; doi:10.1371/journal.pone.0063352)
Supplement: Table S4 — K d values for interactions with the PSD-95 PDZ domains determined by surface plasmon resonance. (PDF) [file pone.0063352.s007.pdf]

Table S4. K_d values for interactions with the PSD-95 PDZ domains determined by surface plasmon resonance.

			K_d (μM)[a,b]		
**Competitor**	**Species**	**Family**	**PDZ1**	**PDZ2**	**PDZ3**
**Reference proteins**					
CRIPT	Human	CRIPT	ND	ND	1.5 ± 0.055
GluN2B	Human	Glutamate gated ion channel	ND	1.4 ± 0.12	ND
KIF1Bα	Human	Kinesin-like	2.2 ± 0.28	1.1 ± 0.16	ND
**G protein-coupled receptors**					
5-HTR_{2A}	Human	5-Hydroxytryptamine receptors	630 ± 12	110 ± 4.3	ND
5-HTR_{2C}	Human	5-Hydroxytryptamine receptors	160 ± 12	62 ± 1.6	780 ± 31
β_1AR	Human	Adrenergic receptors	ND	180 ± 9.4	400 ± 27
β_2AR	Human	Adrenergic receptors	ND	2086 ± 230	ND
BAI1	Human	Class B Orphans	120 ± 3.8	74 ± 3.6	2400 ± 630
Ghrelin	Human	Ghrelin receptor	NA	NA	NA
SSTR1	Human	Somatostatin receptors	84 ± 3.4	76 ± 1.5	ND
SSTR1	Mouse	Somatostatin receptors	270 ± 9.1	ND	ND
SSTR3	Human	Somatostatin receptors	ND	ND	240 ± 7.1

[a]The shown data are K_d ± fitting error.

[b]NA, no affinity. ND, not determined.
